# Assessing Health Care Professionals’ Perceptions of a New System in Clinical Workflows: Systems Engineering Initiative for Patient Safety–Based Consensual Qualitative Research

**DOI:** 10.2196/86166

**Published:** 2026-01-23

**Authors:** Ye-Eun Park, Minsu Ock, Jae-Ho Lee, Dae-Hyun Ko, Hak-Jae Lee, Taezoon Park, Junsang Yoo, Yura Lee

**Affiliations:** 1 Department of Information Medicine Asan Medical Center University of Ulsan College of Medicine Seoul Republic of Korea; 2 Department of Preventive Medicine Ulsan University Hospital University of Ulsan College of Medicine Ulsan Republic of Korea; 3 Department of Emergency Medicine Asan Medical Center University of Ulsan College of Medicine Seoul Republic of Korea; 4 Department of Laboratory Medicine Asan Medical Center University of Ulsan College of Medicine Seoul Republic of Korea; 5 Division of Acute Care Surgery, Department of Surgery Asan Medical Center University of Ulsan College of Medicine Seoul Republic of Korea; 6 Department of Industrial & Information Systems Engineering Soongsil University Seoul Republic of Korea; 7 Department of Digital Health Samsung Advanced Institute for Health Sciences & Technology Sungkyunkwan University Seoul Republic of Korea

**Keywords:** decision support systems (clinical), precision medicine, patient safety, blood transfusion, algorithms (artificial intelligence), information system

## Abstract

**Background:**

Artificial intelligence (AI)–enabled clinical decision support systems (CDSSs) are increasingly embedded within electronic health record (EHR) environments; however, their introduction can disrupt existing workflows and raise patient safety concerns, particularly in high-stakes settings such as surgical transfusion. Limited qualitative evidence exists regarding how frontline professionals anticipate the clinical, organizational, and workflow implications of such systems before wider deployment.

**Objective:**

This study aims to qualitatively examine the anticipated clinical, organizational, and workflow-level implications of implementing personalized Maximum Surgical Blood Order Schedule—Thoracic Surgery (pMSBOS-TS), an AI-enabled CDSS for personalized surgical blood ordering, before large-scale deployment.

**Methods:**

We conducted a consensual qualitative study with 14 multidisciplinary health care professionals involved in transfusion-related tasks at a large tertiary hospital. Following 1 pilot focus group to refine the interview guide and workflow diagram, 2 semistructured focus group discussions were held with 14 participants (5 physicians, 6 nurses, and 3 blood bank staff). Transcripts were analyzed using the Systems Engineering Initiative for Patient Safety (SEIPS) 101 framework, focusing on People, Environment, Tools, and Tasks, and were supported by task- and workflow-based analyses of transfusion processes. Member checking was conducted with participants and external clinicians to enhance validity.

**Results:**

A total of 189 semantic units and 61 core ideas were identified across 18 subdomains and 7 overarching domains. Participants anticipated that pMSBOS-TS could reduce unwarranted variation in blood ordering and planning, provided that algorithmic performance is reliable and the interface is tightly integrated into existing EHR workflows. At the same time, they expressed concerns regarding increased verification burden, system limitations in unexpected clinical scenarios, and potential communication bottlenecks between clinical units and the blood bank. Organizational culture, governance structures, and local transfusion logistics were viewed as critical determinants of whether the system would reduce or inadvertently increase workload and blood product waste.

**Conclusions:**

This preimplementation, SEIPS-based qualitative evaluation suggests that the successful adoption of an AI-enabled transfusion CDSS depends not only on predictive performance but also on sociotechnical readiness, including user trust, workflow fit, and organizational support. These findings provide practice-based insights to inform staged implementation, training, and governance strategies aimed at safely integrating predictive transfusion CDSSs into EHR-supported surgical workflows.

## Introduction

The integration of artificial intelligence (AI) and digital systems into clinical practice is transforming health care delivery. As health care professionals increasingly encounter emerging technologies, their acceptance and perceptions of these systems substantially influence clinical efficiency and implementation success [1-3]. Despite growing interest in digital innovations, including clinical decision support systems (CDSSs), their adoption can disrupt existing workflows, necessitating careful evaluation of organizational- and user-level impacts [4,5].

Although prior workflow analyses have predominantly focused on identifying the root causes of patient safety incidents [6-8], there is an increasing need for proactive assessments during the early stages of system deployment. In particular, the rapid proliferation of electronic health record (EHR)–based applications requires a deeper understanding of how novel systems interface with existing clinical processes. Nevertheless, comprehensive workflow analyses addressing the multifaceted challenges of system adoption remain limited.

The widespread implementation of EHRs, accelerated by initiatives such as the Meaningful Use Program under the Health Information Technology for Economic and Clinical Health Act [9-13], has catalyzed the development of embedded CDSSs to enhance care quality and operational efficiency [14-16]. However, the adoption and integration of such tools remain complex, particularly in high-stakes settings such as surgical transfusion.

Traditional evaluation approaches, such as user satisfaction surveys or system log data, are useful for capturing surface-level feedback but often fall short in explaining context-dependent interactions among health care professionals and between users and systems [17]. Consequently, rigorous qualitative methodologies are essential for understanding the nuanced implications of technology integration [18].

Accordingly, this consensual qualitative research (CQR) study aimed to conduct a preimplementation evaluation of the personalized Maximum Surgical Blood Order Schedule—Thoracic Surgery (pMSBOS-TS) system by examining its anticipated implications for workflows, usability, and organizational conditions, and by identifying factors that may support its safe and effective integration into clinical practice [19]. To our knowledge, only a few studies have examined AI-enabled CDSSs for transfusion planning using a structured preimplementation evaluation; most existing CDSS research has focused on postdeployment clinician acceptance and use rather than prospective workflow and system-integration assessments [20]. By applying CQR alongside a sociotechnical framework before system deployment, this study provides a multistakeholder assessment of how an AI-based transfusion tool may influence workflows, communication patterns, and organizational processes.

## Methods

### System Description (pMSBOS-TS)

pMSBOS-TS is a machine learning–based CDSS developed to generate personalized maximum blood-ordering recommendations for thoracic surgery patients by integrating patient-, laboratory-, and procedure-specific predictors [21]. The underlying algorithms were developed and validated in a prior work by the collaborating investigators [19], and this study evaluates the system’s anticipated effects in a real-world clinical context.

### Study Site

This study was conducted at Asan Medical Center, a 2764-bed tertiary hospital that performs approximately 70,892 surgeries annually as of 2023.

### Study Design

This qualitative study examined the anticipated effects of applying the pMSBOS-TS system for health care professionals involved in transfusion tasks, under the assumption that the system was integrated into the existing electronic medical record (EMR). The assessment used the Systems Engineering Initiative for Patient Safety (SEIPS) framework to evaluate potential impacts across the domains of People, Environment, Tools, and Tasks (PETT) [22,23].

Originally introduced by Carayon et al [22] and subsequently expanded, SEIPS conceptualizes health care as comprising interacting components—person, task, technology/tools, organization, and environment—that shape care processes and, ultimately, outcomes such as patient safety and care quality [23,24]. SEIPS 2.0 incorporated patients and families as active participants and placed greater emphasis on processes, while SEIPS 3.0 expanded the framework to the patient-journey level, including cross-setting transitions.

To enhance accessibility, Holden and Carayon [23] proposed “SEIPS 101,” a simplified, practice-oriented adaptation of the model. SEIPS 101 retains the core elements of the work system, process, and outcomes, while streamlining the work system into 4 primary components (PETT). In this study, *people* refer to health care stakeholders (clinicians and patients) and their capabilities or needs; *tasks* denote activities and workflows; *tools/technology* include equipment, information technology (IT) systems (CDSS/health information systems), and other job aids; and *environment* encompasses both the physical setting (eg, layout, lighting, and noise) and the social or organizational context (eg, culture, policies, and teamwork).

By focusing on these elements and their interactions, a SEIPS/PETT-based evaluation can map how a new CDSS influences clinical work—for instance, how it reshapes clinicians’ tasks, introduces tool-related issues, or alters team dynamics—and how these changes affect care processes and outcomes. This sociotechnical perspective is particularly useful for identifying system-level issues that purely clinical or IT-focused evaluations may overlook [25].

**Figure 1:**
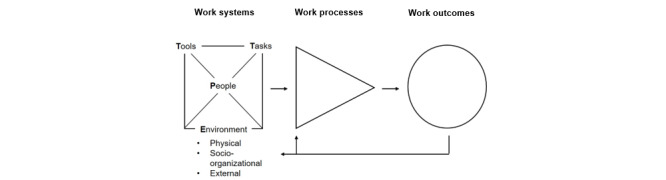
Simplified SEIPS 101 model of work systems, processes, and outcomes. SEIPS: Systems Engineering Initiative for Patient Safety.

A key advantage of this approach is its holistic orientation toward workflow and safety, providing a structured method for analyzing and designing health care processes. The PETT scan can serve as both a checklist and a documentation tool to ensure that all relevant components of the work system are systematically addressed. Accordingly, we applied the PETT scan to identify barriers and facilitators across components and to explore their interactions, thereby providing a comprehensive overview of the work system. Our methodology systematically applied the PETT scan tool from the SEIPS framework to assess users, the surrounding environment, tasks, and tools/technology (Figure 1).

**Figure 2:**
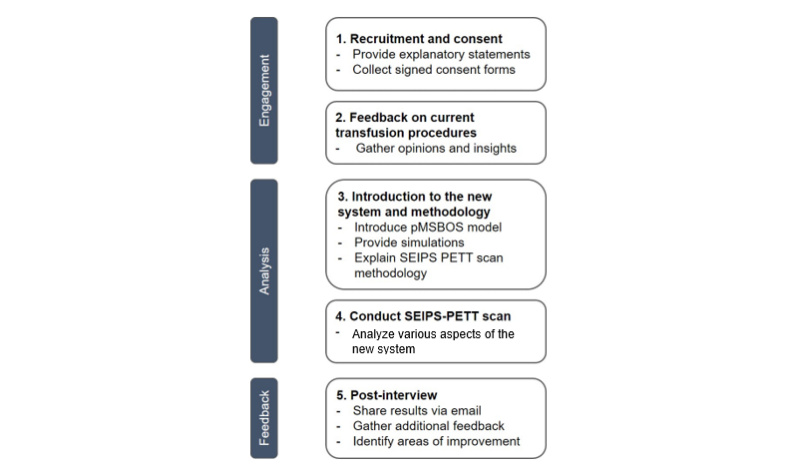
Overview of the participant engagement and SEIPS-PETT analysis process. PETT: People, Environment, Tools, and Tasks; pMSBOS-TS: personalized Maximum Surgical Blood Order Schedule—Thoracic Surgery; SEIPS: Systems Engineering Initiative for Patient Safety.

Three group sessions were conducted between October 19, 2023, and May 2, 2024, following a structured 5-stage process: participant recruitment, workflow feedback, system introduction, PETT-based evaluation, and postsession feedback (Figure 2).

### Recruitment

To assess the usability and workflow implications of the pMSBOS-TS system, we recruited health care professionals currently involved in transfusion-related tasks and identified as potential end users of the system. Recruitment was conducted via the hospital’s online bulletin board and direct outreach. Eligible participants were those with more than 1 year of experience in procedures or surgeries with a high likelihood of requiring transfusion and who had experience handling blood products.

Inclusion criteria were surgeons prescribing blood products, nurses in surgical wards and operating rooms handling blood requests and transfusions, and blood bank staff managing the release and distribution of blood products.

Three focus group discussions (FGDs) were conducted for this study: 1 pilot session and 2 main sessions, each comprising 3-8 participants. Group composition was balanced across roles (eg, surgeons, nurses, and blood bank personnel) to gather diverse perspectives while maintaining thematic consistency. A semistructured interview guide was used for all sessions, and each participant took part in only 1 session.

### Data Collection

All FGDs were conducted in person in a private meeting room within the hospital. No individual interviews were conducted; all qualitative data were collected through FGDs. Each session was facilitated by YL (female), a trained moderator with expertise in qualitative health research, and assisted by YEP (female), who managed logistics, note-taking, and audio recording. Although the researchers and participants worked within the same institution, no direct personal or supervisory relationships existed. Participants were informed of the study objectives and were aware that the moderator was a member of the research team; no personal goals or interests of the researchers were disclosed.

A semistructured interview guide was used in all sessions. Each session lasted 60-80 minutes and was audio-recorded with participant permission. Participants were encouraged to describe specific cases and reflect on workflow steps using printed workflow diagrams and sticky-note categorization. All participants were invited to speak, write, categorize notes, and present their views during the sessions.

All interviews were conducted in Korean, transcribed verbatim, reviewed by participants, and subsequently translated into English. Translation was performed by bilingual researchers fluent in both Korean and English. To ensure semantic equivalence, translated transcripts were reviewed by a second bilingual researcher, and discrepancies were resolved through discussion. To minimize interpretive bias, participants were emailed the FGD transcripts organized by content unit to verify accuracy and identify any distortions or omissions in meaning. No participants had a prior personal relationship with the moderator.

The methodology and reporting of the qualitative findings followed the COREQ (Consolidated Criteria for Reporting Qualitative Research) guidelines [26]. We adhered to the COREQ guidelines in describing the study design, data collection, and qualitative analysis procedures.

### Qualitative Analysis

#### Overview

The qualitative analysis was conducted in accordance with the principles of CQR to minimize interpretive distortion and to derive in-depth insights from clinical field experts regarding the use of pMSBOS-TS [27].

#### Initial Stage of Qualitative Analysis: Preliminary Discussions and Hypothesis Formation

The research team hypothesized that personalized prediction could support more efficient use of blood products. Based on a literature review of the adoption of new medical information systems and expert discussions, we concluded that, in addition to understanding the risks and benefits of pMSBOS-TS, factors related to its successful implementation would be essential [28,29].

Accordingly, the following core questions were developed:

- “What positive or negative impacts could be expected if pMSBOS-TS is introduced into the clinical field?”
- “What factors are important for the successful adaptation and implementation of pMSBOS-TS in clinical practice?”

To conduct a multidimensional evaluation of the risks and impacts associated with introducing a new application, we employed the PETT scan framework. Prior research using the SEIPS model was reviewed to inform the development of the interview guide [22,23]. Participants actively engaged with the PETT framework by manually categorizing insights using sticky notes, which facilitated recall and reflection. They also participated in a transfusion-related workflow analysis to identify potential impacts at each step.

A pilot focus group session was conducted with clinical professionals to examine transfusion-related processes in greater detail and to identify clinical scenarios relevant to the application of pMSBOS-TS. This pilot session served multiple purposes: it validated the structure and appropriateness of the PETT-based interview methodology, supported the development of a draft transfusion workflow diagram, and informed the refinement of the initial domain-subcategory-core idea table. Although the pilot session provided useful foundational insights, its data were excluded from the final cross-case analysis to ensure consistency with the main dataset.

#### Intracase Analysis: Task Analysis and Development of Domains and Subdomains

To ensure comprehensive capture of participants’ views on the impacts and potential harms of pMSBOS-TS at each stage of the transfusion process, a detailed task analysis was performed [30,31]. The analysis team, comprising 2 PhD-level researchers and 1 research assistant with a bachelor’s degree, developed the following domains, subdomains, and core ideas based on the core questions:

- Domains: transfusion-related work experiences (case examples), pMSBOS-TS–related opinions, and major categories derived from the PETT scan.
- Subdomains: specific subcomponents of the PETT scan and preliminary workflow elements identified during the pilot focus group sessions.
- Core ideas: derived by reviewing interview transcripts and postinterview notes; preliminary categorizations were validated through consultation with 2 domain experts.

#### Cross-Case Analysis

Two analysis team members (YEP and YL) independently reviewed and coded meaningful units from the transcripts and sticky notes into subdomains and core ideas. Coding discrepancies were resolved through consensus discussion or, when necessary, adjudicated by a third team member. Core ideas that did not fit existing categories were temporarily placed in an “Other” category and redefined as needed.

A frequency-based coding scheme was employed to reflect both sentiment and the prevalence of opinions: positive opinions were marked with plus signs (+), and negative opinions with minus signs (–). Frequency was indicated as follows: 1 symbol for 1 participant, 2 symbols for 2-3 participants, and 3 symbols for 4 or more participants. Expression formats and interpretation strategies were discussed and agreed upon for divergent opinions within the same theme.

Categories were defined as follows:

- General: common across all sessions (– – –/+ + +).
- Typical: consistent within a session but not endorsed by all participants.
- Variant: mixed opinions or views that appeared in only a subset of cases.

For validation, the finalized core ideas were shared via email with the original participants for member checking to ensure accuracy and minimize interpretive distortion. In addition, 3 clinical professionals from other medical institutions (meeting the same inclusion criteria as the interview participants) were contacted. After explaining the study objectives and methodology, the core ideas and task-analysis flow diagram were shared via email to confirm that the interpretations were neither biased nor incomplete. Data saturation was assessed during cross-case analysis; no new core ideas emerged from experts outside the interview group, indicating thematic saturation consistent with CQR guidelines.

Themes and core ideas were derived inductively from the data in accordance with the CQR approach, whereas the overarching domains were organized using the SEIPS/PETT framework. No themes were predetermined before analysis.

### Ethics Statement

This study was approved by the Institutional Review Board (IRB) of Asan Medical Center, Korea (IRB 2023-0724), and conducted in accordance with relevant ethical guidelines. Informed consent was obtained from all participants before their involvement, with assurances of anonymity and confidentiality. Participants were briefed on the study’s objectives and the intended use of the collected data. Participants received compensation for their participation.

## Results

### Participants

Fourteen participants took part in 2 FGDs, comprising 5 physicians (3 fellows/professors and 2 residents), 6 nurses (4 from the surgical intensive care unit, 1 from the anesthesia and recovery unit, and 1 from the internal medicine ward), and 3 blood bank staff members (Table 1). Detailed participant characteristics, including participant identifiers, are provided in Multimedia Appendix 1.

**Table 1:** Demographics of the participants.

Variables		Doctor (n=5)	Medical technologist (n=3)	Nurse (n=6)	Total (N=14)
**Sex, n**					
	Male	2	1	0	3
	Female	3	2	6	11
Age, mean (SD)		30 (11)	40 (14.1)	28.3 (6.9)	31.4 (11.7)
Transfusion experience, mean (SD)		8.2 (9.4)	20.3 (10.1)	13 (7.2)	13.53 (10.12)
**Interviews participated in, n**					
	First	2	3	3	8
	Second	3	0	3	6

We organized the results of a task analysis of transfusion-related processes using a swim-lane approach (Multimedia Appendix 2). From the workflow analysis and FGDs, we extracted 189 unique semantic units (81 in category A and 108 in category B, excluding duplicates). These units were organized according to their relevance to transfusion workflows. Semantic units describing inefficient use or cases involving blood products, regardless of whether pMSBOS-TS was applied, were classified as category A. By contrast, statements concerning anticipated impacts of pMSBOS-TS implementation or potential risks associated with its use were classified as category B.

The semantic units were further condensed into 61 core ideas: 21 from category A and 40 from category B. These were then organized into 18 subdomains (3 in category A and 15 in category B) and 7 overarching domains (1 in category A and 6 in category B). The complete analytical framework, including domains, subdomains, and core ideas, is summarized in Multimedia Appendix 3.

In category A, titled “(In)Efficient Use of Blood Components,” 3 subdomains were identified: *Inaccurate Dosage (Over- or Under-Dosing), Inefficient Blood Usage and Processes,* and *Other Transfusion Process Errors.*

Category B comprised 6 primary domains: *People, Environment, Tools, Tasks, Work Processes*, and *Work Outcomes*. Within the People domain, 2 subdomains were identified: *Prescribing Physicians* (3 general and 1 variant core idea) and *Other Transfusion Stakeholders* (1 general and 1 variant core idea). The Environment domain included 2 subdomains: *Interprofessional Communication* and *Socio-organizational Context*. The Tools domain encompassed aspects related to algorithmic performance, interface design, and perceived reliability. The Tasks domain included *Achieving Algorithm Objectives*, *Task Complexity and Variability*, and *Handling Unanticipated Situations*.

In the Work Processes domain, responses were classified based on tasks associated with the application phase of pMSBOS-TS (eg, order entry and related processes). The Work Outcomes domain addressed participants’ perceptions of the consequences or results associated with the algorithm’s use.

### Category A: Notable Experiences in Transfusion Practice or Cases of Inefficient Blood Product Use

Of the 21 core ideas from category A, 6 were classified as *typical* and another 6 as *variant*. Within the subdomain “Inaccurate Dosage (Over- or Under-Dosing),” 2 typical and 2 variant core ideas were identified. Participants primarily highlighted experiences related to the prescribing and preparation phases (Table 2).

Regarding unintentional over- or underprescription, several participants referred to habitual or standardized prescribing practices in their departments that led to unnecessary transfusions.

We routinely prescribed three units of RBCs, even when we did not actually end up transfusing them...

Participant 2_5

A view that routine surgeries rarely require transfusions was also expressed. During the preparation stage, including order verification on the ward and confirmation/preparation in the blood bank, participants noted discrepancies between prescribed and requested volumes.

When a patient is going into surgery, they often pre-prescribe fresh frozen plasma, and the blood bank will call us saying it was too much and ask to cancel it. It is a tough situation...

Participant 1_8

By contrast, a blood bank staff member pointed out that excessive orders often stemmed from physicians’ personal preferences:

Sometimes the physician asks us to save much more blood than needed. So we end up placing unnecessary orders. It makes our inventory look abundant to the central blood center, but when we actually have an emergency, there is not much usable blood left.

Participant 1_2

In the subdomain of “Inefficient Blood Usage and Processes,” 2 typical and 2 variant core ideas were identified. Participants noted issues in communication and procedural transparency during the transfusion preparation process.

I often feel there is a disconnect between prescription and preparation. There is a timing issue when blood is prepared, and we are supposed to call the physician directly—but sometimes we miss it. If the doctor does not say, “I have prescribed blood for tomorrow,” we will not know until we ask. Then they just say, “Do not worry, it is for tomorrow.” But in those communication gaps, we sometimes fail to have the blood ready when the patient actually needs it.

Participant 1_4

In addition, logistical difficulties in transporting blood products within the hospital were cited, including challenges related to elevators, distance, and location.

Sometimes we cannot even get an elevator. I think it is pretty hard for the ward staff to go pick up the blood themselves. We end up waiting a long time for the blood to arrive...

Participant 1_4

Some participants also described inconveniences in transfusion process workflows, including managing low blood inventory levels, emergency dispatch of blood transport vehicles, and cumbersome steps such as cross-matching or additional compatibility testing.

In the subdomain of “Other Transfusion Process Errors,” 2 typical and 1 variant core ideas were identified. These primarily involved unexpected clinical events during procedures. For example, some participants described scenarios in which unanticipated vascular damage occurred during surgery.

One that really stands out is during CS or liver transplant surgeries—suddenly the aorta or artery tears, and we are hooking up multiple blood bags to the massive transfusion machine. It is just intense during those massive transfusions.

Participant 1_6

Other participants reflected on communication breakdowns and disorganized processes during emergencies:

Nobody is talking to each other, everyone is busy...Trying to get consent from the caregiver, some people are preparing the transfusion, others are trying to set up IV lines for massive transfusion, even when the lines are not ready yet—they just keep trying anyway...

Participant 1_5

**Table 2:** Cross-case analysis for inefficient use of blood components (category A).

Domain, subdomain, and core idea				Frequency^a^					Total frequency			
				Group 1		Group 2						
1. Efficient Use of Blood Components												
	**1.1. Inaccurate Dosage (Over-/Underdosing)**											
		1.1.1. Prescription: clinician-driven over- or underordering	+ +		0		Variant					
		1.1.2. Prescription: over- or underordering not based on individual discretion	+		+ +		Typical					
		1.1.3. Preparation: over-/underpreparation during ward request or blood bank confirmation	+ +		+		Typical					
		1.1.4. Administration: clinically unnecessary or inadequate transfusions	+		0		Variant					
	**1.2. Inefficient Blood Usage and Processes**											
		1.2.1. Lack of transparency in communication or procedures during transfusion preparation	+		+ +		Typical					
		1.2.2. Issues in subprocess tasks for order fulfillment	+		+		Typical					
		1.2.3. Logistics issues in internal transport of blood products	+ +		0		Variant					
		1.2.4. Blood management challenges	+ +		0		Variant					
		1.2.5. Preparation of transfusion support devices	0		0		N/A^b^					
		1.2.6 Blood product wastage	0		0		N/A					
	**1.3. Other Transfusion Process Errors**											
		1.3.1. Patient identity and data checks	0		0		N/A					
		1.3.2. Transfusion ordering	0		+		Variant					
		1.3.3. Order verification	0		0		N/A					
		1.3.4. Ward‑level blood preparation	+		0		Variant					
		1.3.5. Blood bank unit preparation	0		0		N/A					
		1.3.6. Blood transport and arrival confirmation	0		0		N/A					
		1.3.7. Transfusion administration	+		+		Typical					
		1.3.8. Transfusion completion	0		0		N/A					
		1.3.9. Other: unforeseen clinical scenarios	+ +		+		Typical					

^a^Frequency indicators reflect both sentiment and prevalence of opinions. Positive opinions are denoted by plus signs (+) and negative opinions by minus signs (–). One symbol indicates 1 participant, 2 symbols indicate 2-3 participants, and 3 symbols indicate 4 or more participants. “0” indicates that no relevant statements were identified for that core idea.

^b^N/A: not applicable (also see footnote “a”).

### Category B: Perceptions and Opinions Regarding pMSBOS-TS

#### People

Regarding the impact of pMSBOS-TS on individual users, participants generally agreed that the system could reduce variation in blood ordering volumes attributable to the prescribing physician’s personal tendencies (Table 3).

We used to just prescribe based on our assumptions, but now this app (pMSBOS-TS) prescribes based on what it studied through machine learning.

Participant 2_5

Reduced deviation according to the characteristics of the physicians’ prescribing tendencies (stable/adventurous).

Sticky note

**Table 3:** Cross-case analysis for the 6 primary domains: People, Environment, Tools, Tasks, Work Processes, and Work Outcomes (category B).

Domain, subdomain, and core idea					Frequency^a^							Total frequency
					Group 1			Group 2				
**1. People**												
	**1.1. Prescribing Physicians**											
		1.1.1. Variability in prescribed volume based on the physician’s experience or skillset (may decrease variation [P^b^] or increase it [N^c^])	+ +			+ +			General			
		1.1.2. Adaptation gap according to the physician’s proficiency with the ordering system	+ +			+ +			General			
		1.1.3. Clinician perceptions of CDSS^d^ affecting adoption of the new system	+ +			+ +			General			
		1.1.4. Final verification of calculated transfusion requirement and prescribing responsibility must remain with the ordering physician	0			+ + +			Variant			
	**1.2. Other Transfusion Stakeholders**											
		1.2.1. Blood bank staff, nurses, and other transfusion team members gain improved demand forecasting through the system	+ +			+ + +			General			
		1.2.2. Other: potential to reduce nonuser‑initiated over‑ or underordering	0			+			Variant			
**2. Environment**												
	**2.1. Interprofessional Communication**											
		2.1.1. Clear communication between clinical staff and the blood bank is critical for successful system adoption	+ +			0			Variant			
		2.1.2. System may enhance transparency (P) but could introduce additional confirmation steps or confusion (N)	– – –			– –			General			
	**2.2. Socio‑Organizational Context**											
		2.2.1. Organization’s culture around individual variation in transfusion demand: blame culture may decrease (P), or lack of clear norms may increase confusion (N)	– –			+			Variant			
		2.2.2. Organizational climate and policies influence uptake	+ +			+ +			General			
		2.2.3. Institutional blood management challenges affect implementation	+ +			+			Typical			
		2.2.4. Clarity of governance is critical for program sustainability	+			+ +			Typical			
		2.2.5. Other: variations in system impact and blood management practices expected based on health care facility size and infrastructure	0			+			Variant			
	**2.3. Physical Environment**											
		2.3.1. Anticipated effective utilization in settings with high concentrations of clinical staff and resources	+			0			Variant			
		2.3.2. Physical environment factors are critical for successful adoption	+			0			Variant			
**3. Tools**												
	**3.1. Algorithm Performance**											
		3.1.1. System response time is a key determinant for adoption	0			+ + +			Variant			
		3.1.2. Ability to incorporate a wide range of clinical input variables	+ +			+ + +			General			
		3.1.3. Capability to generate tailored recommendations for various blood components	+			+ + +			Typical			
		3.1.4. Predictive accuracy of the algorithm is essential	+ +			+ +			General			
	**3.2. Usability and System Design**											
		3.2.1. Interface layout and input mechanisms must support efficient use	+ +			+ +			General			
		3.2.2. Workflow integration should not interrupt clinical tasks	+			+			Typical			
		3.2.3. Seamless electronic medical record interoperability to prepopulate patient data and eliminate manual entry	+ +			+ + +			General			
		3.2.4. Other: support for operation across diverse platforms	+			0			Variant			
	**3.3. Trust in the Tool**											
		3.3.1. Confidence in the pMSBOS-TS^e^ model is critical for sustained use	+ +			+			Typical			
		3.3.2. Other: difficulty establishing confidence in the algorithm due to the inherently opaque machine‑learning inference process	0			+			Variant			
**4. Tasks**												
	**4.1 Achieving Algorithm Objectives**											
		4.1.1. Impact on returns/wastage: personalized demand forecasts should reduce waste (P) or, if overestimation/mistrust occurs, increase waste (N)	+			+/– – –			Variant			
		4.1.2. Supply from the National Blood Service: procurement may become easier (P) or more difficult (N)	–			0			Variant			
		4.1.3. Other: improved blood inventory management may help reduce delays in transfusion	0			+			Variant			
	**4.2. Task Complexity and Variability**											
		4.2.1. System introduction may decrease (P) or increase (N) required time and effort	+/–			++/–			Variant			
		4.2.2. Must accommodate complex cases	+ +			0			Variant			
	**4.3. Handling Unanticipated Situations**											
		4.3.1. Flexibility to manage unforeseen variables not captured by the algorithm	+ +			+ + +			General			
		4.3.2. Impact from overlapping work‑system issues	+ +			+ +			General			
		4.3.3. Limiting tool application to defined scenarios ensures safe and effective use	0			+ +			Variant			
		4.3.4. Other: malfunctions or unintended consequences may arise if users do not fully understand the task or intended use of the system.	+			0			Variant			
**5. Work Processes**												
	**5.1. Linked Diagnostic Orders**											
		5.1.1. Integration of ancillary tests with the transfusion program is critical	+			+ +			Typical			
	**5.2. Preparation of Related Procedures/Equipment**											
		5.2.1. System may facilitate preparation of downstream tasks (P) or, conversely, increase complexity of subsequent steps (N)	–			+ +/– –			Variant			
**6. Work Outcomes**												
	**6.1. Efficiency of Blood Use and Management**											
		6.1.1. Contribution of pMSBOS-TS to efficient utilization and inventory control: positive or negative	+ +			+/– – –			Variant			
		6.1.2. Long‑term impact: accumulation of usage data to further refine predictive accuracy and inform adoption strategies	+			+ +			Typical			
		6.1.3. Indirect clinical benefits: supports expedited detection of abnormal laboratory findings, thereby enhancing overall patient management	+			+ +			Typical			
	**6.2. Organizational Culture and Processes for Personalized Transfusion**											
		6.2.1. Role of pMSBOS-TS in fostering a culture and workflow for personalized maximum transfusion prediction: positive impact or none/negative impact	– –			–			Typical			

^a^Frequency indicators reflect both sentiment and prevalence of opinions. Positive opinions are denoted by plus signs (+) and negative opinions by minus signs (–). One symbol indicates 1 participant, 2 symbols indicate 2-3 participants, and 3 symbols indicate 4 or more participants. “0” indicates that no relevant statements were identified for that core idea.

^b^P: positive.

^c^N: negative.

^d^CDSS: clinical decision support system.

^e^pMSBOS-TS: personalized Maximum Surgical Blood Order Schedule—Thoracic Surgery.

However, it was also noted that the effectiveness of pMSBOS-TS would depend on the prescriber’s level of proficiency with the system and their trust in its recommendations.

Someone who judges based on his/her own experience and is not familiar with a new tool, may pursue the existing method.

Sticky note

In one group, it was emphasized that final confirmation and responsibility for blood ordering based on calculated transfusion requirements should remain with the physician. Among the broader group of health care professionals involved in the transfusion process, there was a general expectation that pMSBOS-TS would support more accurate predictions of required blood volumes.

#### Environment

Regarding the environmental impact of pMSBOS-TS, participants expressed concern that its implementation might lead to increased verification steps or procedural confusion during blood preparation and release.

There used to be a set standard of 3 or 2 (example of the number of blood packs in order set), but if it changes for each patient...like, this patient needs to prepare 8, this patient needs to prepare 5...If our nurses had to check with the prescribing doctor each time and prepare a certain number of bloods, I thought there could be confusion.

Participant 1_5

There was also a shared perception that the success of integration would depend heavily on the organizational culture and internal dynamics of the institution.

I think that the surgical nursing team may focus more on the team atmosphere when the pMSBOS-TS is implemented.

Participant 2_3

Opinions were divided between the 2 groups regarding whether pMSBOS-TS would positively or negatively influence the development of an organizational culture that accommodates interindividual variability in transfusion needs.

#### Tools

Regarding pMSBOS-TS as a tool, participants broadly agreed that the algorithm should be capable of incorporating a wider range of clinical input variables, and that its predictive performance—specifically, its accuracy—would be crucial to successful adoption.

Even for the same disease, the bleeding risk differs depending on the severity, and since previous abdominal surgery has a big influence on the tissue adhesion, it seems likely that more blood transfusions will be needed. I wonder to what extent this will be reflected in the algorithm.

Participant 1_7

With respect to usability, participants consistently emphasized that the interface should be intuitive, with screen layouts and input controls designed for ease of use. They also stressed that patient information should be automatically integrated from the EMR to minimize manual data entry by users.

If (the data input of pMSBOS-TS is) not linked to EHR, additional workload is possible/input error is possible.

Sticky note

The importance of system response speed was highlighted frequently in only 1 of the 2 groups.

#### Tasks

Regarding the interaction between pMSBOS-TS and task performance, participants generally agreed that the system’s ability to flexibly accommodate unexpected clinical scenarios or variables not accounted for by the algorithm would be critical.

Variation in the skill of the surgeon performing the surgery/The possibility of an unexpected worse situation occurring for the patient.

Sticky note

Task performance was also noted to be influenced when issues from other components of the broader work system intersected with the use of pMSBOS-TS. Opinions were divided across both groups regarding the anticipated impact of pMSBOS-TS on blood product returns or waste, as well as on the complexity and performance of transfusion-related tasks.

#### Work Processes and Work Outcomes

No dominant themes emerged concerning the influence of pMSBOS-TS on work processes or outcomes. Opinions were mixed regarding whether the system would affect subsequent tasks or contribute meaningfully to the management and utilization of blood products.

The pMSBOS-TS would be helpful in assessing the appropriateness of blood transfusion (for example, for health insurance’s claim eligibility review)

Participant 1_1

If more blood is prescribed to account for the risk of bleeding, there may be more problems with distribution, such as returns or disposal.

Participant 2_6

## Discussion

### Principal Findings

This study identified clinicians’ anticipated benefits and concerns regarding pMSBOS-TS across workflow, usability, and organizational domains. Consistent with prior CDSS research, such systems can offer clear benefits when successfully implemented, enhancing patient safety and supporting clinical decision-making. Through a qualitative investigation involving frontline health care professionals, we explored anticipated impacts—both positive and negative—associated with implementing pMSBOS-TS, a prediction-based CDSS for maximum surgical blood ordering in preoperative patients.

Findings revealed several core concepts that extend beyond transfusion tasks and are broadly applicable to CDSS implementation. These include the importance of users’ (ie, physicians’) system proficiency and trust, the influence of organizational culture, the accuracy of task performance, and overall system usability. By engaging diverse stakeholders and applying the PETT framework, we examined the hypothesis that successful and safe CDSS implementation depends not only on algorithmic performance but also on workflow integration and interprofessional interactions.

### Interpretation and Implications

These observations illustrate how pMSBOS-TS may interact with existing transfusion workflows and sociotechnical structures. Whereas traditional MSBOS approaches are often static (eg, “for procedure X, always have Y units ready”), machine learning models can personalize recommendations by incorporating patient-specific factors [19]. Given the urgent and collaborative nature of transfusion processes, participants noted concerns regarding potential unintended effects on interdisciplinary collaboration and emphasized the need for flexibility within the system’s functionality. These insights suggest that, for a newly introduced CDSS to achieve its intended impact, attention must be paid not only to algorithmic performance but also to the specific characteristics of the task environment, end users, and interface design.

Task analysis has long been used to address patient safety issues and guide quality improvement initiatives in health care, including the design, implementation, and optimization of health IT systems [30,32,33]. During the development of the swim-lane diagram, we observed that transfusion workflows extend beyond a simple physician-patient interaction, involving complex coordination among physicians, nurses, and blood bank staff. Findings from category A confirmed that inefficiencies arise not only from interprofessional issues but also from interdepartmental and spatial constraints. Compared with medication administration, transfusion processes are more resource-constrained and require specialized procedures—matching, delivery, storage, and multistep verification. As a result, discrepancies in prescribing or preparation can lead to significant resource waste and additional workload. Moreover, blood banks require specialized expertise and must coordinate directly with central blood suppliers, making communication breakdowns a potential source of operational strain.

This complexity was reflected in the cross-case analysis for category A, which identified inefficient practices such as over- or underprescribing influenced by order sets or department-specific habits. These findings align with participants’ views that organizational culture will play a central role in shaping the success of pMSBOS-TS implementation. Concerns regarding inconsistent blood preparation, lack of transparency, and potential confusion during rollout further underscore the importance of attention to workflow readiness. Additionally, the need for compatibility testing, highlighted in both categories A and B, emphasizes that evaluations must account for the entire sequence of related workflows, including upstream preparation and downstream follow-up.

The core question—“What positive or negative impacts could be expected if pMSBOS-TS is introduced into clinical practice?”—elicited both supportive and critical responses. Anticipated benefits included reduced prescription variability and improved accuracy of blood use predictions, aligning with the system’s development goals. However, participants expressed divergent views regarding downstream tasks, such as adjusting over- or underprescriptions driven by order sets and managing transfusion-related tools. Differences also emerged regarding the expected benefits of personalized blood ordering—short term (eg, time savings and reduced blood waste) versus long term (eg, improved blood reserve management).

The most frequently cited concern was the potential for increased workload due to additional confirmation (double-checking) steps. Given that transfusions often occur in life-threatening situations, this emphasis on safety is understandable. The introduction of a confirmation mechanism is an essential safeguard, and participants’ concerns reflect a broadly shared and appropriate level of caution. In addition, several participants noted that patient-specific transfusion considerations—such as previous alloimmunization, allergic reactions, or rare blood requirements—are not represented in the current algorithm and would continue to require clinical judgment. These factors delineate important boundaries for safe use and underscore the need for guidance on when algorithmic recommendations should be supplemented by clinician expertise.

Participants also voiced skepticism regarding the impact of personalized services on clinical workflows, a sentiment commonly reported in the adoption of precision medicine tools. This resistance may partly stem from the lack of distinction between short- and long-term effects in the interview questions. As with many new technologies, early implementation often increases user workload. Thus, realizing the intended benefits of systems such as pMSBOS-TS requires not only user engagement but also effective communication with stakeholders, workflow adaptation, and careful integration into existing processes.

In response to the question, “What factors are important for the successful adaptation and implementation of pMSBOS-TS in clinical practice?,” participants commonly cited human and environmental factors, such as clinician trust and organizational culture, alongside functional considerations, including input/output variables, interface usability, and EMR integration. These findings align with prior research identifying such factors as critical to successful system adoption [28,29]. Given that transfusions often occur in emergencies, such as massive intraoperative bleeding or rapid clinical deterioration, participants emphasized the need for system flexibility and attention to overlapping workflows.

These insights suggest that successful implementation requires not only optimization of algorithmic performance and user-centered interface design but also enhanced user training and a supportive organizational environment. System adoption is not linear but cyclical and interactive, involving system use, feedback, and iterative refinement. Accordingly, the relationship between algorithms and workflows should be understood as bidirectional.

Furthermore, adaptation should be evaluated in stages during system implementation. Conflicting opinions regarding expected effects—such as concerns about increased confirmation steps or confusion during blood withdrawal or preparation—reflect common apprehensions in clinical settings. This underscores the need for ongoing, structured efforts to minimize unintended consequences and ensure effective integration into routine workflows.

Although participants received preinterview materials and program demonstrations, many required further clarification, particularly regarding how personalized transfusion calculations could reduce overall prescription volume. The machine learning processes underlying these calculations were frequently described as a “black box,” and comprehension remained limited even with explainable AI functions. Although participants commonly perceived the model as a black box, not all machine learning approaches lack transparency; for example, tree-based or regression-based models may provide interpretable feature contributions, though such details were beyond the scope of this preimplementation evaluation [21]. This highlights the need for further research on clinician education, comprehension, and acceptance when introducing AI-driven clinical tools.

Implementing a novel AI-driven CDSS in a complex health care environment is not merely a technical deployment—it represents a sociotechnical change that reverberates through clinicians’ routines, tasks, and organizational structures. A SEIPS-based evaluation captures this full spectrum of impacts by examining PETT across each stage of the workflow, enabling identification of both anticipated benefits and unanticipated drawbacks [23]. Although other analytic approaches provide useful insights, SEIPS offers a unifying systems perspective that is particularly valuable for complex, high-risk interventions such as CDSSs in clinical care. We also emphasize the importance of balancing approaches by supplementing SEIPS findings with quantitative measures from prior studies and by ensuring practical applicability through the simplified SEIPS 101 model, which facilitates stakeholder engagement [19,23].

### Strengths and Limitations

This study has several limitations. It was conducted in a single country and institution, and our FGD findings—derived from the SEIPS framework and task analysis—are qualitative and context-specific, which may limit replicability. In addition, the SEIPS framework has been criticized for its complexity and limited applicability to macro-level evaluation [23]. To mitigate these concerns, we performed content validation with medical professionals from external institutions who were not involved in the interviews. Given the characteristics of tertiary hospitals, some findings may not generalize to smaller institutions with fewer personnel dedicated to transfusion management. Nevertheless, because regular transfusion-related surgeries are less common in smaller hospitals and transfusion practices involve multiple specialties, future work should include a broader range of settings and professional roles.

As a result of the FGD-based task analysis and qualitative design, recall bias and incomplete reporting are possible. Follow-up research (eg, system usage log analyses) could address these limitations; however, such data were not available for this preimplementation evaluation of a new CDSS [34]. This may represent a common constraint in predeployment CDSS impact or risk assessments. Future studies should complement qualitative findings with quantitative, log-based evaluations as these data become available.

As we targeted actively practicing medical professionals, the physicians in our sample had shorter career durations than other groups. This reflects the staffing structure of the clinical environment and is unlikely to affect the analysis of practical workflows. However, future research should examine how professional seniority may influence perceptions related to organizational decision-making and AI governance.

Given the specific nature of transfusion workflows, some requirements identified in this study may not generalize to other AI applications. Moreover, to accurately assess the impact of new systems, future evaluations should differentiate expected effects across stages of system adoption and adaptation. This limitation became apparent during our analysis and warrants attention in subsequent research. Although successful AI deployment depends heavily on the human-system interface, current literature lacks theory-driven qualitative evaluations examining how AI fits within complex sociotechnical systems. We addressed these challenges by engaging diverse stakeholders, applying a structured sociotechnical framework, and adhering to rigorous qualitative research methods.

### Overall Contribution

This study contributes to CDSS implementation research by providing one of the few preimplementation evaluations of an AI-based transfusion decision support tool. Using CQR and the SEIPS 101 framework, we captured practical concerns and anticipated impacts across physicians, nurses, and blood bank staff, offering concise, context-specific insights that complement findings from prior postdeployment CDSS studies.

### Conclusions

As systems and workflows interact dynamically, it is essential to consider both tool performance and contextual adaptation. Future research should segment the adaptation process and examine interactions among users, their roles, and organizational dynamics.

## Appendix

Multimedia Appendix 1 Demographic and professional characteristics of focus group discussion participants.

Multimedia Appendix 2 Integrated workflow for preoperative blood ordering, preparation, and transfusion in surgical care settings.

Multimedia Appendix 3 Analytical framework, including domains, subdomains, and core ideas.

## Abbreviations

AI: artificial intelligence
CDSS: clinical decision support system
COREQ: Consolidated Criteria for Reporting Qualitative Research
CQR: consensual qualitative research
EHR: electronic health record
EMR: electronic medical record
FGD: focus group discussion
IRB: institutional review board
IT: information technology
PETT: People, Environment, Tools, and Tasks
pMSBOS-TS: personalized Maximum Surgical Blood Order Schedule—Thoracic Surgery
SEIPS: Systems Engineering Initiative for Patient Safety

## Data Sharing

The datasets generated and analyzed during this study are not publicly available due to the lack of prior consideration for data sharing during study design and Institutional Review Board approval. However, data are available from the corresponding author (YL) upon reasonable request and with approval from the Asan Medical Center Institutional Data Access/Ethics Committee.
