# Supplementary material for: Assessing Health Care Professionals’ Perceptions of a New System in Clinical Workflows: Systems Engineering Initiative for Patient Safety–Based Consensual Qualitative Research
Source: J Med Internet Res. 2026 Jan 23;28:e86166. doi: 10.2196/86166 (PMC12881895; doi:10.2196/86166)
Supplement: Multimedia Appendix 1 [file jmir_v28i1e86166_app1.docx]

Multimedia Appendix 1. Demographic and Professional Characteristics of FGD Participants.

| FGD Group | Participant No. | Sex | Age | Length of career (clinical field) | Affiliation |
| --- | --- | --- | --- | --- | --- |
| 1 | 1‒1 | Female | 50s | 28 years | Blood bank |
|  | 1‒2 | Male | 50s | 27 years | Blood bank |
|  | 1‒3 | Female | 20s | 6 years | Blood bank |
|  | 1‒4 | Female | 20s | 5 years | Surgical Intensive Care Unit nurse |
|  | 1‒5 | Female | 20s | 2 years | Internal medicine ward nurse |
|  | 1‒6 | Female | 30s | 17 years | Anaesthesia and recovery unit nurse |
|  | 1‒7 | Male | 20s | 4 years | Resident |
|  | 1‒8 | Female | 20s | 2 years | Resident |
| 2 | 2‒1 | Female | 30s | 18 years | Surgical Intensive Care Unit nurse |
|  | 2‒2 | Female | 30s | 14 years | Surgical Intensive Care Unit nurse |
|  | 2‒3 | Female | 40s | 22 years | Surgical Intensive Care Unit nurse |
|  | 2‒4 | Female | 50s | 27 years | Professor |
|  | 2‒5 | Male | 30s | 4 years | Fellow |
|  | 2‒6 | Female | 30s | 4 years | Fellow |
