# Supplementary material for: Assessing Health Care Professionals’ Perceptions of a New System in Clinical Workflows: Systems Engineering Initiative for Patient Safety–Based Consensual Qualitative Research
Source: J Med Internet Res. 2026 Jan 23;28:e86166. doi: 10.2196/86166 (PMC12881895; doi:10.2196/86166)
Supplement: Multimedia Appendix 2 [file jmir_v28i1e86166_app2.docx]

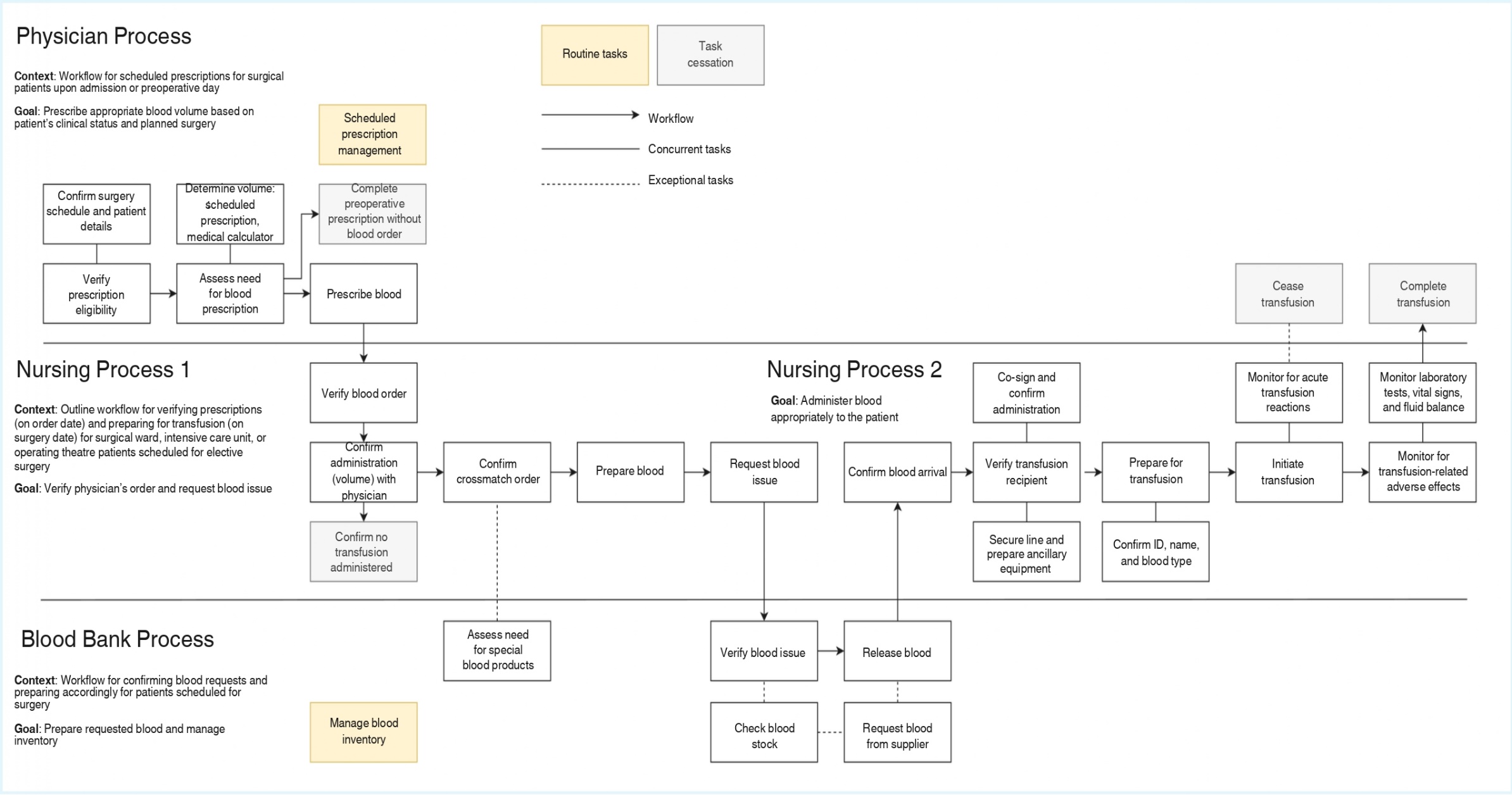


Multimedia Appendix 2. Integrated Workflow for Preoperative Blood Ordering, Preparation, and Transfusion in Surgical Care Settings.

Presented as a swim lane diagram illustrating the distinct roles and sequential tasks of physicians, nurses, and the blood bank. The workflow depicts prescription verification, determination of blood volume, processing of blood requests, and transfusion administration for patients scheduled for elective surgery in surgical wards, intensive care units, or operating theatres. Context annotations describe the clinical scenarios assumed during process design, including prescription verification on the day of ordering, preparation on the day of surgery, and confirmation of blood requests in advance of scheduled procedures. Arrows indicate workflow sequences, solid lines denote concurrent tasks, and dotted lines denote exceptional tasks. No error bars are present in this figure. Abbreviations: ID, identification; pMSBOS, personalized Maximum Surgical Blood Order Schedule
