# Supplementary material for: Assessing Health Care Professionals’ Perceptions of a New System in Clinical Workflows: Systems Engineering Initiative for Patient Safety–Based Consensual Qualitative Research
Source: J Med Internet Res. 2026 Jan 23;28:e86166. doi: 10.2196/86166 (PMC12881895; doi:10.2196/86166)
Supplement: Multimedia Appendix 3 [file jmir_v28i1e86166_app3.docx]

Multimedia Appendix 3. Analytical framework, including domains, subdomains, and core ideas.

| **Category** | **Domain** | **Sub-domain** | **Core Idea** |
| --- | --- | --- | --- |
| A | 1. Efficient Use of Blood Components | 1.1. Inaccurate Dosage (Over-/Under-Dosing) | 1.1.1. Prescription: clinician-driven over- or underordering (such as inadequate patient assessment, habitual prescribing bias) |
|  |  |  | 1.1.2. Prescription: over- or underordering not based on individual discretion (such as standing orders, specialty-specific routines) |
|  |  |  | 1.1.3. Preparation: over-/underpreparation during ward request or blood bank confirmation |
|  |  |  | 1.1.4. Administration: clinically unnecessary or inadequate transfusions (such as transfusion in low-need cases, lack of proper patient evaluation) |
|  |  | 1.2. Inefficient Blood Usage Processes | 1.2.1. Lack of transparency in communication or procedures during transfusion preparation |
|  |  |  | 1.2.2. Issues in subprocess tasks for order fulfilment (such as blood draw for cross‑match) |
|  |  |  | 1.2.3. Logistics issues in internal transport of blood products |
|  |  |  | 1.2.4. Blood management challenges (such as delays in rare blood procurement, stock balancing, returns/re‑allocation of over-prepared units) |
|  |  |  | 1.2.5. Preparation of transfusion support devices (such as massive transfusion set‑ups, filters) |
|  |  |  | 1.2.6 Blood product wastage (such as over‑ordering/preparation, expiry, delays in transfusion decision‑making) |
|  |  | 1.3. Other Transfusion Process Errors | 1.3.1. Patient identity and data checks (such as lab results, medication history, procedure type) |
|  |  |  | 1.3.2. Transfusion ordering (such as system input mistakes, inappropriate use of order sets or calculators) |
|  |  |  | 1.3.3. Order verification |
|  |  |  | 1.3.4. Ward‑level blood preparation (matching, issuing requests) |
|  |  |  | 1.3.5. Blood bank unit preparation |
|  |  |  | 1.3.6. Blood transport and arrival confirmation |
|  |  |  | 1.3.7. Transfusion administration (patient verification, line preparation) |
|  |  |  | 1.3.8. Transfusion completion (infusion end, monitoring for reactions) |
| **Category** | **Domain** | **Sub-domain** | **Core Idea** |
| B | 1. People | 1.1 Prescribing Physicians | 1.1.1. Variability in prescribed volume based on the physician’s experience or skillset (may decrease variation [P] or increase it [N]) |
|  |  |  | 1.1.2. Adaptation gap according to the physician’s proficiency with the ordering system |
|  |  |  | 1.1.3. Clinician perceptions of CDSS (bias, attitude, trust) affecting adoption of the new system |
|  |  |  | 1.1.4. Final verification of calculated transfusion requirement and prescribing responsibility must remain with the ordering physician |
|  |  | 1.2 Other Transfusion Stakeholders | 1.2.1. Blood bank staff, nurses, and other transfusion team members gain improved demand forecasting through the system |
|  | 2. Environments | 2.1 Inter‑Professional Communication | 2.1.1. Clear communication between clinical staff and the blood bank is critical for successful system adoption |
|  |  |  | 2.1.2. System may enhance transparency (P) but could introduce additional confirmation steps or confusion (N) |
|  |  | 2.2 Socio‑Organizational Context | 2.2.1. Organization’s culture around individual variation in transfusion demand: blame culture may decrease (P) or lack of clear norms may increase confusion (N) |
|  |  |  | 2.2.2. Organizational climate and policies (such as the chest surgery department’s stance on pMSBOS-TS) influence uptake |
|  |  |  | 2.2.3. Institutional blood management challenges (such as shortages; delayed rare units; inventory coordination; returns/reallocation) affect implementation |
|  |  |  | 2.2.4. Clarity of governance (record‑keeping; responsible department) is critical for program sustainability |
|  | 3. Tools | 3.1 Algorithm Performance | 3.1.1. System response time is a key determinant for adoption |
|  |  |  | 3.1.2. Ability to incorporate a wide range of clinical input variables (such as patient comorbidities, hemodynamic status, lab values) |
|  |  |  | 3.1.3. Capability to generate tailored recommendations for various blood components (such as RBCs, platelets, plasma) |
|  |  |  | 3.1.4. Predictive accuracy of the algorithm is essential |
|  |  | 3.2 Usability and System Design | 3.2.1. Interface layout and input mechanisms must support efficient use |
|  |  |  | 3.2.2. Workflow integration (notifications; prompts) should not interrupt clinical tasks |
|  |  |  | 3.2.3. Seamless EMR interoperability to prepopulate patient data and eliminate manual entry |
|  |  | 3.3 Trust in the Tool | 3.3.1. Confidence in the pMSBOS-TS model is critical for sustained use |
|  | 4. Tasks | 4.1 Achieving Algorithm Objectives | 4.1.1. Impact on returns/wastage: personalized demand forecasts should reduce waste (P) or, if overestimation/mistrust occurs, increase waste (N) |
|  |  |  | 4.1.2. Supply from the National Blood Service: procurement may become easier (P) or more difficult (N) |
|  |  | 4.2 Task Complexity and Variability | 4.2.1. System introduction may decrease (P) or increase (N) required time and effort |
|  |  |  | 4.2.2. Must accommodate complex cases (such as rare blood types; irradiated products) |
|  |  | 4.3. Handling Unanticipated Situations | 4.3.1. Flexibility to manage unforeseen variables not captured by the algorithm (such as surgeon skill variability) |
|  |  |  | 4.3.2. Impact from overlapping work‑system issues (such as inadequate communication in rare‑blood scenarios; simultaneous high task loads) |
|  |  |  | 4.3.3. Limiting tool application to defined scenarios (such as elective surgeries) ensures safe and effective use |
|  | 5. Work Processes | 5.1 Linked Diagnostic Orders | 5.1.1. Integration of ancillary tests (such as compatibility testing) with the transfusion program is critical |
|  |  | 5.2 Preparation of Related Procedures/Equipment | 5.2.1. System may facilitate preparation of downstream tasks (such as massive transfusion set readiness) (P) or, conversely, increase complexity of subsequent steps (N) |
|  | 6. Work Outcomes | 6.1 Efficiency of Blood Use & Management | 6.1.1. Contribution of pMSBOS-TS to efficient utilization and inventory control: positive or negative |
|  |  |  | 6.1.2. Long‑term Impact: accumulation of usage data to further refine predictive accuracy and inform adoption strategies |
|  |  |  | 6.1.3. Indirect clinical benefits: supports expedited detection of abnormal laboratory findings, thereby enhancing overall patient management |
|  |  | 6.2 Organizational Culture and Processes for Personalized Transfusion | 6.2.1. Role of pMSBOS-TS in fostering a culture and workflow for personalized maximum transfusion prediction: positive impact or none/negative impact |
